# An Update on Cancer Cluster Activities at the Centers for Disease Control and Prevention

**DOI:** 10.1289/ehp.9021

**Published:** 2006-11-30

**Authors:** Beverly S. Kingsley, Karen L. Schmeichel, Carol H. Rubin

**Affiliations:** National Center for Environmental Health, Centers for Disease Control and Prevention, Atlanta, Georgia, USA

**Keywords:** cancer, cancer clusters, Centers for Disease Control and Prevention, environmental hazards, epidemiologic cluster investigations, state health departments

## Abstract

The Centers for Disease Control and Prevention (CDC) continues to be aware of the need for response to public concern as well as to state and local agency concern about cancer clusters. In 1990 the CDC published the “Guidelines for Investigating Clusters of Health Events,” in which a four-stage process was presented. This document has provided a framework that most state health departments have adopted, with modifications pertaining to their specific situations, available resources, and philosophy concerning disease clusters. The purpose of this present article is not to revise the CDC guidelines; they retain their original usefulness and validity. However, in the past 15 years, multiple cluster studies as well as scientific and technologic developments have affected cluster science and response (improvements in cancer registries, a federal initiative in environmental public health tracking, refinement of biomarker technology, cluster identification using geographic information systems software, and the emergence of the Internet). Thus, we offer an addendum for use with the original document. Currently, to address both the needs of state health departments as well as public concern, the CDC now *a*) provides a centralized, coordinated response system for cancer cluster inquiries, *b*) supports an electronic cancer cluster listserver, *c*) maintains an informative web page, and *d*) provides support to states, ranging from laboratory analysis to epidemiologic assistance and expertise. Response to cancer clusters is appropriate public health action, and the CDC will continue to provide assistance, facilitate communication among states, and foster the development of new approaches in cluster science.

Disease clusters continue to concern the public, and public sentiment that environmental causes are responsible and must be investigated is widely prevalent. More than a decade ago, the Centers for Disease Control and Prevention (CDC) recognized the need to develop operating procedures for response to public concern about disease clusters. The National Conference on Clustering of Health Events was held 15–16 February 1989 in Atlanta, Georgia; the proceedings were published (Rothenberg et al. 1990a); and the CDC released the “Guidelines for Investigating Clusters of Health Events” (CDC 1990) in which a four-stage process was presented: *a*) an initial response to gather source information, *b*) an assessment of the occurrence of the health event, *c*) a feasibility study, and *d*) an epidemiologic investigation. During the last 15 years, these guidelines have provided a framework that most state health departments have adopted, modifying it for their specific situations and available resources. The states have the primary responsibility for response to cancer cluster concerns within their domain. The CDC guidelines emphasize the need for health agencies to develop an approach that maintains community relations while responding to clusters efficiently; the approaches vary among states as well as according to the nature of the cluster and the availability of case and comparison data. The orientation of each state-based inquiry response and investigation plan is shaped by state philosophy and experience with previous clusters.

The purpose of this article is not to revise the CDC guidelines; they retain their usefulness and validity. However, in the past 15 years, numerous cluster studies [Agency for Toxic Substances and Disease Registry (ATSDR) 2006; Cochise County Health Department (CCHD) 2005; Heath 2005; Massachusetts Department of Public Health (MDPH) 2005; National Cancer Institute (NCI) 2005; New Jersey Department of Health and Senior Services (NJDHSS) 2004; Rubin et al. 2007] as well as scientific and technologic developments have affected cluster science and response. Thus, we offer an addendum for use with the original document. Included in this list of significant developments are improvements in cancer registries, a federal initiative in environmental public health tracking (EPHT), refinement of biomarker technology, new cluster identification and geographic information systems (GIS) software, and the emergence of the Internet. These developments have shaped the approach of the CDC National Center for Environmental Health (NCEH) for public health response to cancer clusters.

## Role of the CDC/NCEH in Cancer Cluster Response

Over the past several decades, industrialization and urban growth have increased human exposure to numerous toxic substances, and as a result, concern has been raised about their relationship to the etiology of chronic disease. The association between environmental factors and disease was validated in recent studies demonstrating that environmental factors such as tobacco smoke, toxic chemicals, dietary habits, and viral infections significantly increase the risk for several types of cancer (Lichtenstein et al. 2000; Thomas and Karagas 1996). A call for increased attention directed toward investigation of environmental exposure as a cause of chronic disease has been widely voiced in the media, the political establishment, and scientific forums.

NCEH defines a cancer cluster as a greater-than-expected number of cancer cases that occurs within a group of people in a geographic area over a defined period of time. In 2000, representatives from the CDC Division of Cancer Prevention and Control (DCPC), National Institute for Occupational Safety and Health (NIOSH), ATSDR, and NCEH met and recognized the importance of a centrally coordinated cancer cluster response to inquiries within the CDC. They assigned this responsibility to NCEH. The rationale for this decision was the strong public perception that environmental exposures are directly responsible for cancer. NCEH now provides a centralized coordinated response system for cancer cluster inquiries received by the CDC. Since the inception of this new responsibility, NCEH has initiated several cluster-related activities (Table 1).

### Cancer Cluster Public Inquiry Triage System

**Figure 1:**
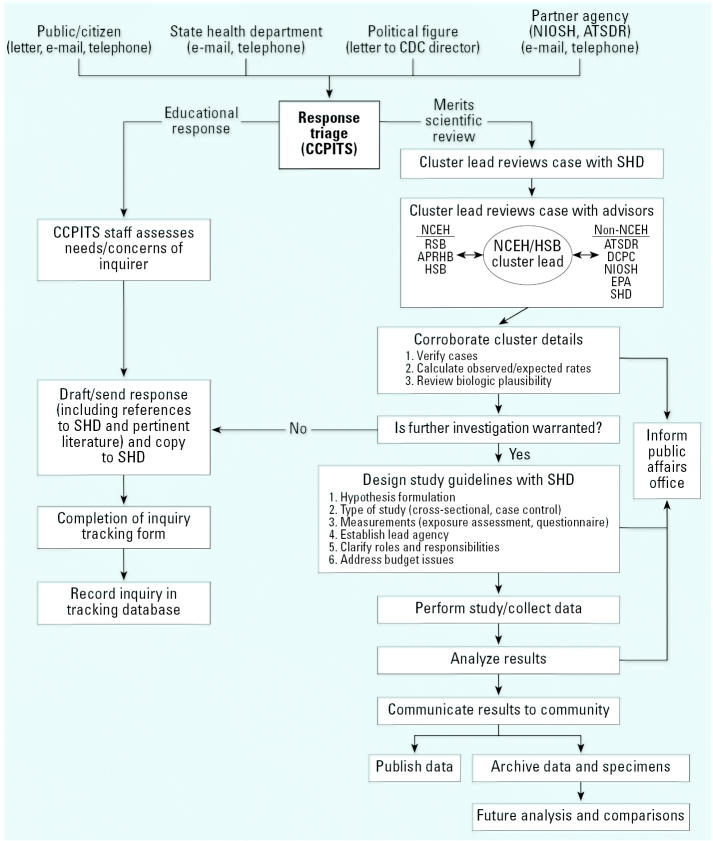
NCEH Cancer Cluster Public Inquiry Triage System. Abbreviations: APRHB, Air Pollution and Respiratory Health Branch; HSB, Health Studies Branch; RSB, Radiation Studies Branch; SHD, state health department; U.S. EPA, U.S. Environmental Protection Agency.

In 2002, NCEH initiated a centralized inquiry system within the CDC, the Cancer Cluster Public Inquiry Triage System (CCPITS; CDC 2004) for responding to cancer cluster inquiries from various audiences such as individual citizens, state health departments, and other federal agencies (Figure 1). The goals of the system are to *a*) provide individual, targeted responses to the public in a timely manner, *b*) decrease the chances of an inquirer having to contact multiple agencies, and *c*) increase communication about cancer clusters among the participating agencies. The response from NCEH to individuals includes basic information on cancer clusters, response to the specific cancer cluster concern, referral to the appropriate state health department and state cancer registry contacts, and links to additional information. NCEH also notifies the state contacts of the inquiry and response. The NCEH cancer cluster website (http://www.cdc.gov/nceh/clusters; CDC 2004) was developed and designed to support the inquiry system and to facilitate information sharing among federal, state, and local agencies; it provides the cornerstone for the cancer cluster inquiry system. If the inquiry concerns a hazardous waste site, work site, or basic cancer issue, NCEH triages the inquiry to ATSDR, CDC/NIOSH, or DCPC, respectively. NCEH tracks inquiry information via an ACCESS database (Microsoft Corp., Redmond, WA) and communication among CDC programs, federal, state, and local agencies is improved as a result of this single point of contact. Since the inception of the system through July 2005, the cancers of concern most frequently cited by inquirers were breast cancer, leukemia, brain cancer, lung cancer, and colorectal cancer. NCEH uses this information proactively to develop additional tools to better serve the needs of inquirers.

When contacted about a potential cancer cluster, NCEH defers to state health departments to provide the first level of response. States examine their cancer registry data, enabling comparison between incidence rates at various geographic levels. State agencies are in the best position to do this because they maintain data on population demographics, local health and environmental issues, and previous investigations. NCEH becomes involved when state health departments request assistance. Several have requested assistance from NCEH pertaining to cancer clusters. NCEH response has ranged from consultation with appropriate staff to active participation in an epidemiologic or biosampling investigation. In some cases, NCEH has provided assistance by conducting analysis of biological samples and storing them for future study, as it did in the childhood leukemia clusters in Churchill County, Nevada (CDC 2003a; Rubin et al. 2007) and Sierra Vista, Arizona (CCHD 2005).

### Media survey of cancer cluster reports

**Figure 2:**
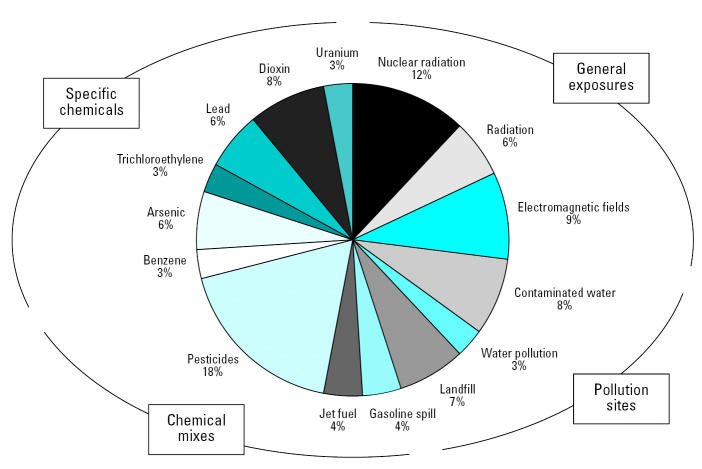
The 15 most commonly used environmental exposure terms found in articles pertaining to cancer clusters published in U.S. newspapers from 1977 to 2001.

Because the media plays a large role in shaping public perception of cancer as well as community concerns about cancer, NCEH conducted a descriptive study of cancer cluster reports in the popular media and characterized the media reports retrieved. A systematic search of newspaper articles on cancer clusters was performed using DIALOG (http://www.dialog.com/sources/subject) and NEXIS (https://www.lexis.com/research) databases. Media reports were categorized according to the specific incident reported and characterized across several variables, including date, state, number of citations, and perceived environmental exposure (Figure 2). The search produced a media record database containing 1,440 records of approximately 175 suspected cancer cluster reports for the period 1977 through 2001. These data reflect the environmental complexities associated with cancer cluster epidemiology as well as the breadth of popular concern and awareness regarding issues of exposure types, pollution sites, and specific environmental chemicals.

In attempting to report on issues of importance to the public, such as the relationship of apparent disease clusters to environmental exposures, the media may occasionally and perhaps unwittingly misrepresent scientific issues and information (Blake 1995; Nelkin 1996). The attempt of the media to focus on human interest, conflicting information, blame, and political symbolism may not always be conducive to the presentation of correct and unbiased information (Greenberg and Wartenberg 1990). In addition the relationship between the environment and disease, and the science underlying cluster investigation (e.g., definition of geographic boundaries, calculation of expected rates of disease, population demographics, etiology of cancer, statistical issues) are complex topics and can be difficult for persons without scientific education and training to comprehend (Turney 1996). Thus, the media, whether electronic, print, or other format, has both a critical role and a significant opportunity in the presentation of information surrounding cluster investigations.

### State-specific activities

In 2001, NCEH conducted a survey to assess state protocols for responding to cancer cluster inquiries and state criteria for conducting investigations. This survey was part of an effort to define and describe existing state-based activities concerning suspected cancer clusters, identify gaps in current investigation methods, and examine opportunities for increasing the efficiency and utility of state and federal efforts. The survey instrument, developed by NCEH, was distributed to 56 states and territories (50 states plus the District of Columbia, Guam, American Samoa, Puerto Rico, the Federated States of Micronesia, and the U.S. Virgin Islands). Although the results of the survey indicated considerable variation among protocols, as well as their perspectives and experiences in cancer cluster investigations, every state or territory that completed the survey (89% participation rate) provided education concerning cancer and/or cancer clusters to all inquirers as part of their inquiry response. Criteria commonly used to determine whether to proceed toward a more intense level of investigation of a cluster included

- identification of a single cancer type;
- biological plausibility and adequate latency; for the reported cancer;
- political pressure;
- identification of a common cancer in an unusual age group;
- identification of a rare cancer;
- identification of exposure to a carcinogenic substance;
- elevated ratio of observed/expected confirmed cancer cases.

To understand further the experiences of state health departments during recent cancer cluster investigations, NCEH conducted site visits to four states (New Jersey, Arizona, Massachusetts, Ohio) in which there were in-depth investigations of leukemia in circumscribed areas, documented protocols for handling cluster inquiries, and considerable experience and expertise in the investigation of disease clusters.

NCEH also sponsored two workshops during which representatives from 10 states (Massachusetts, Florida, California, South Carolina, Montana, New York, Georgia, Minnesota, Texas, and Washington) met and discussed cancer cluster activity. From these workshops and site visits, several conclusions were drawn. All states and territories placed a high importance on educational components and provided education to all callers. In addition most had standardized forms to facilitate information gathering, took a systematic approach, triaged incoming inquiries, were interested in improved science and methodology, followed the framework suggested by the 1990 CDC guidelines (CDC 1990), and had cancer cluster websites. However, response varied greatly depending upon state experience and political considerations. All states and territories were well aware of the inherent complexities in cancer cluster investigations, including data quality, migration, latency, small numbers, and political issues. Most protocols were continuing to evolve.

The states and territories listed several needs during the workshops:

- validation from a federal agency;
- additional funding and personnel;
- training (e.g., CDC-sponsored workshops on methods and media relations);
- CDC-facilitated information/data sharing;
- assistance with complex investigations.

Details of the workshop proceedings may be found at http://www.cdc.gov/nceh/clusters/cluster_response.htm (CDC 2003b).

In 2003 in response to a recommendation voiced at the workshop, NCEH established an electronic listserver (CDC 2003c) to facilitate dialogue among the states and federal agency staff and to provide a mechanism to share and discuss data and scientific methods.

Beginning in 2001, the CDC became involved in two childhood leukemia cluster investigations at the request of state and local health departments. As part of these two cross-sectional exposure assessments in Churchill County, Nevada (CDC 2003a; Rubin et al. 2007), and Sierra Vista, Arizona (CCHD 2005), the CDC conducted detailed laboratory analyses of biological samples.

## Recent Environmental Health Initiatives That Impact Cluster Response

Public demand for the investigation of the relationship between environmental exposure and disease has produced a number of significant technologic and programmatic advancements, especially in data collection (cancer/chronic disease registries), data quality (exposure analysis and biomonitoring), and data analysis (statistical methods in spatiotemporal analysis and GIS). Progress in each of these areas has the potential to improve the efficacy of future cancer cluster studies.

### Data collection

Registries provide useful information to estimate disease incidence and prevalence, evaluate epidemics and disease spread, monitor control and prevention measures, detect changes in health practices, recognize newly emerging diseases, maintain a historical archive of data, and facilitate epidemiologic research (Koo et al. 2005; Macdonald et al. 1996; Teutsch and Thacker 1995; Thacker 1994). Implementation of surveillance measures also serves to increase public confidence in the commitment of the government to protect public health (Hertz-Picciotto 1996).

Although national disease registries and surveillance programs were initially designed for infectious disease monitoring, the use of registries for chronic disease surveillance has gained increased popularity and funding over the last several decades. (Berkelman and Buehler 1990; Koo et al. 2005; Thacker et al. 1995). To detect elevated chronic disease levels and to implement appropriate prevention programs, it is important to establish a baseline for disease occurrence. This requires extensive registry-based databases about disease occurrence under “usual” conditions (Rothenberg et al. 1990b). An increasing number of registries have been established to track chronic adverse health effects, with cancer registries emerging as a gold standard in chronic disease surveillance. (Koo et al. 2005; McGeehin et al. 2004; Thacker and Stroup 1996).

The NCI initiated the Surveillance Epidemiology and End Results (SEER) program in 1973 to serve as an authoritative source of information on cancer incidence and survival in the United States (NCI 2006a). SEER now provides cancer surveillance for 26% of the U.S. population (Wingo et al. 2003). In 1992 the U.S. Congress passed the Cancer Registries Amendment Act (1992), which called for the enhancement of existing statewide cancer registries and authorized a centralized cancer registry program to cover those states not enrolled in SEER. As a result of this congressional act, the CDC implemented the National Program of Cancer Registries (NPCR; http://www.cdc.gov/cancer/npcr/index.htm; CDC 2006). NPCR complements SEER programs by supporting statewide cancer registries in 45 states, the District of Columbia, and three U.S. territories. Together, SEER and NPCR cover approximately 96% of the U.S. population (Wingo et al. 2003). Registry certification for SEER and NPCR is provided by the North American Association of Central Cancer Registries (NAACCR 2006), which provides guidance to all state registries to achieve data content and compatibility levels acceptable for pooling data and improving national estimates. Since the publication of the 1990 guidelines (CDC 1990), state and regional cancer registries have developed significantly with respect to registry numbers, capacity, and standardization. As a result, cancer registry data will provide high-quality, timely information to support response to concerned communities and facilitate proactive early detection and remediation, especially for cancers with short latency periods (Elliott and Wartenberg 2004; Koo et al. 2005; Raymond 1989; Thacker 1994).

An important initiative that will impact the work of the CDC in cancer clusters is the development of a national EPHT network. In September 2000, the Pew Environmental Health Commission recommended the creation of a coordinated public health system to track diseases and environmental exposures and to identify environmental health threats (Environmental Health Tracking Project Team 2000). In response, the U.S. Congress funded the CDC in 2002 to begin the development of an EPHT network that would link information on environmental hazards, human exposures to those hazards, and health effects potentially related to those hazards (McGeehin et al. 2004). The goal of this initiative is to integrate hazard monitoring, exposure surveillance, and health effects surveillance into a cohesive national EPHT network. Information gained from this network can be used to respond to and reduce the burden of environment-related disease. Partners in this effort include federal, state, and local health and environmental agencies, nongovernmental organizations, academic institutions, health care organizations, and community groups and members. As a result of this program, geographic areas and populations likely to be affected by environmental contamination will be identifiable, and communities can be provided with valuable information on their environment and potential health implications.

### Data quality

Until recently, measurements of human exposure to toxins were usually extrapolated from data collected by environmental sampling, personal interview, or exposure modeling (Lioy 1995; Sexton et al. 1992). Efforts have recently shifted toward the development of a wide array of biological markers that can directly determine the portion of a given chemical that enters the body and at what level it can cause damage or disease (Decaprio 1997; Sampson et al. 1994; Sexton et al. 2004). Biomarkers of exposure, effect, and susceptibility are currently measured in a variety of biological substances, including blood (serum and peripheral blood lymphocytes), urine, breast milk, feces, adipose tissue, hair, nails, semen, exhaled breath, and buccal, nasal, or bronchial epithelia (Barr and Needham 2002; Sexton et al. 2004).

Approximately 300 chemicals can currently be assayed by the NCEH Division of Laboratory Sciences (CDC 2005a), including polychlorinated biphenyls, dioxins, persistent and nonpersistent organic pesticides and their metabolites, polyaromatic hydrocarbon metabolites, metals, volatile organic compounds, and phytoestrogens. As part of the National Health and Nutrition Examination Survey (NHANES), the public health survey conducted by the CDC National Center for Health Statistics (Sexton et al. 2004), biological samples collected from thousands of U.S. residents are tested for numerous environmental chemicals. The resulting data are used to compile the *CDC National Report on Human Exposure to Environmental Chemicals*. This report is published every 2 years; the 2005 report includes 148 compounds (CDC 2005b). NHANES biomonitoring provides baseline exposure data for a number of substances not previously monitored and has been an important comparative tool in the assessment of chemical exposures.

### Data analysis: available software and calls for guidance

An appendix to the CDC 1990 disease cluster guidelines presented an exhaustive summary of available methods to test for spatial and/or temporal clustering (CDC 1990). Although these methods remain valid and actively used in cluster investigations, many have limited application in cancer cluster analysis because of the associated complexities: long latency period, multiple exposures, genetic susceptibility, migration, small area investigations with limited case numbers, and data quality and resolution (Elliott and Wartenberg 2004; Jacquez 2004; Kulldorff and Hjalmars 1999; Wakefield and Elliott 1999; Wartenberg 2001). Recognizing these limitations, scientists have refined existing methods and developed additional ones. The topic has been the subject of lively discussion in the literature (Cuzick and Edwards 1990; Jacquez et al. 1996b; Waller 2000; Wartenberg 1995; Wartenberg and Greenberg 1993). Several national and international workshops have also addressed this issue: the 1992 Workshop on “Statistics and Computing in Disease Clustering” in Port Jefferson, New York; the 1994 Conference on “Statistics and Computing in Disease Clustering” sponsored by the NCI in Vancouver, British Columbia, Canada; and the 1997 World Health Organization “Disease Mapping and Risk Assessment for Public Health” in Rome, Italy (Jacquez et al. 1993, 1996a; Lawson et al. 2000; Wakefield et al. 2001). As a result of the increased intensity in the field of cluster statistics, more than 100 analytic methods are currently available (NAACCR 2002).

A number of software programs and packages, from both private and public sectors, have been developed to service the needs of the public health cluster response community (Table 2). The choice of method is determined by ease of use, user familiarity, and funds available for software, updates, and training (Jacquez et al. 1996b; Wartenberg 2001; Wartenberg and Greenberg 1993). To address the issue of appropriate use of methodology in cancer cluster analysis, expert panels have been organized and have provided concise and practical guidance on choice of clustering methods and software. For example, during the 2002 North American Association of Central Cancer Registries GIS workgroup meeting in Princeton, New Jersey, organized by NAACCR, the GIS subcommittee called for a systematic review of currently available, open-access cluster analysis software programs for cancer registries (NAACCR 2004). The discussion resulted in the publication of a detailed and systematic comparison of four cluster software packages (Crimestat, SaTScan, R-Geo, and GeoDa) (Anselin 2004).

Methodologic guidance was also the impetus for a 2002 NCI meeting on “Current Practices in Spatial Data Analysis” in Bethesda, Maryland, convened to focus specifically on spatial data analysis. Proceedings from this meeting were published in three journal articles on cluster methodology that summarize the state of the art, discuss ongoing limitations, reinforce important caveats, and envision future developments (Boscoe et al. 2004; Jacquez 2004; Pickle et al. 2005). Increased attention to cancer cluster methodologies represents a significant advancement in its own right; however, published recommendations for method selection vary, and the need for extensive systematic comparisons of different approaches remains (Jacquez 2004; Wartenberg 1995; Wartenberg and Greenberg 1993). Although some studies have already examined circumstance-appropriate applications for these methods (Hill et al. 2000; Song and Kulldorff 2003), additional studies of this nature should allow for clearer guidelines on methods usage for the epidemiologist and other disease cluster investigators.

### Combining cluster methodologies with GIS

Cluster analysis has been further bolstered by the incorporation of GIS into the cancer cluster approach. Basic GIS tools are now being used routinely to depict and display potential cancer clusters in visually compelling ways, for example, the NCI Atlas of Cancer Mortality for the United States (http://www3.cancer.gov/atlasplus/; NCI 2006b). Recently, more complicated applications for GIS have included the active surveillance of cancer data to detect cancer clustering, a venture requiring coordinated implementation of GIS mapping and statistics-based spatiotemporal cluster analyses (Muravov et al. 1998; Rushton 2003; Sheehan et al. 2000).

However, unlike infectious diseases, which are relatively limited in time and space, chronic diseases such as cancer create analytical complexities not readily accommodated by current conventional GIS techniques and software packages (Jacquez 2004; Jarup 2004). Moreover, issues concerning the accuracy and quality of data, metadata standards, inconsistent resolution of data points, aggregated data usage, and issues of privacy and confidentiality have yet to be fully resolved (Elliott and Wartenberg 2004; Jacquez 2004; Melnick and Fleming 1999; Nuckols et al. 2004; Waller 2000).

Extensive discussions about the use of GIS in disease cluster analyses were held at the 1998 National Conference on GIS in Public Health in San Diego, California (ATSDR 1998; Richards et al. 1999) and the 2002 North American Association of Central Cancer Registries GIS Workgroup meeting in Princeton, New Jersey (NAACCR 2002). Critics of the current use of GIS in cluster analysis note that this technology was originally developed for business purposes to generate static “snapshots” of locational information (Thrall 1999). They suggest that this approach would be more useful in investigating the etiology of chronic diseases if it were reengineered to evaluate geographic locations in a dynamic time series (Jacquez 2004; Sabel et al. 2000). Thus, while GIS technology gains momentum in cluster investigations and is increasingly used for cancer registries and by cluster responders, new developments such as including more systematic approaches to cluster investigations, improved accuracy in data acquisition, and consideration of genetic susceptibility may be necessary to achieve optimal results (Jacquez 2004; Pickle et al. 2005; Richards et al. 1999).

## Conclusions

Cancer cluster investigations occasionally have led to the discovery of important pathways in the etiology of specific cancers, such as with angiosarcoma (Waxweiler et al. 1976), lung cancer (U.S. Department of Health Education and Welfare 1964), Kaposi sarcoma (CDC 1981, 1982), vaginal clear-cell carcinoma (Herbst et al. 1971), bladder cancer (Villanueva et al. 2003), and scrotal cancer (Pott 1775). However, it is important to note that the majority of these studies that yielded etiologic information were studies of occupational, drug-induced, or infectious pathogenic exposure rather than studies of environmental exposure (Caldwell 1990; Heath 2005). Nonetheless, in some cases, geographic clusters with suspected environmental etiology warrant follow-up (Wartenberg 2001). Cluster response is appropriate public health practice, but resources must be used efficiently and wisely. Responses to cluster inquiries include addressing community concerns, providing community education, and informing the community about the progress of investigations and/or exposure assessments.

Recently, a paradigm shift in cluster response has taken place: as analytic methods improve, exposure assessment using biologic sampling is now more commonly employed as part of the public health response (Decaprio 1997). Consequently, significant environmental exposure information may be obtained as well as an increased potential for detection of environment and disease relationships. Now that baseline human exposure data are available from the “Third National Report on Human Exposure to Environmental Chemicals” (CDC 2005b), results from biomonitoring investigations may be much more meaningful.

The experience of federal, state, and local public health agencies with cancer cluster concerns and investigations has demonstrated the influence that mass media may have on the community concerning health, disease, and the environment. Although the underlying science of cancer cluster investigations is complex, the importance of providing this information to the public in a clear, balanced, and scientifically correct format cannot be overstated.

## Future Directions

The link to environmental exposures, whether perceived or actual, is an important issue that must be addressed. The many similarities across states in their efforts to respond to cancer cluster inquiries create the opportunity for state and federal agencies to better coordinate their efforts. An important role for the CDC is, and will continue to be, to facilitate communication among states and others, to provide assistance when appropriate, to provide increased access to relevant data, and to foster the development of new systems, tools, and approaches to cluster investigation.

## Figures and Tables

**Table 1:** Recent CDC-sponsored cancer cluster activities in the United States.

Year	CDC cluster activities
2002	CCPITS
2002–2003	Survey of state protocols in cancer clusters (56 states/territories)
2002	State site visits (AZ, OH, NJ, MA)
2003	State and Federal Technical Capacity Building Workshop: Response to Cancer Clusters with Suspected Environmental Etiology (CA, FL, GA, MA, MD, MN, NY, SC, TX, WA; CDC 2003b)
2003	Electronic listserver (185 participants; CDC 2003c)
2002–2003	Survey of media reports on cancer clusters
2001–2003	Assistance to Nevada State Department of Health; Cross Sectional Exposure Assessment of Case Children with Leukemia and a Reference Population in Churchill County, Nevada (CDC 2003a; Rubin et al. 2007)
2003–2005	Assistance to the CCHD and the Arizona Department of Health Services; Biosampling of Children with Leukemia plus a Comparison Population in Sierra Vista, Arizona (CCHD 2005)

**Table 2:** Software available for disease cluster analysis.

Software name/package	Fee	GIS functions	Website	Reference
CLUSTER 3.1	Yes	None	http://www.atsdr.cdc.gov/HS/cluster.html	ATSDR 2005; Hall et al. 1996
ClusterSeer	Yes	Compatible	http://www.terraseer.com/products/clusterseer.html	Jacquez 1996; TerraSeer, Inc. 2006
CrimeStat 3.0	Yes	None	http://www.icpsr.umich.edu/NACJD/crimestat.html	Levine 2004
DMAP^a^	No	Built in	http://www.uiowa.edu/~gishlth/DMAP/	University of Iowa 1997
EpiAnalyst	Yes	Compatible	http://www.phrl.org/REGS/Info%20EpiAnalyst.htm	Research Epidemiology Geographic Software 2003
GeoDa 0.9.5-1^a^	No	Built in	http://www.geoda.uiuc.edu/default.php	Anselin 2003; Anselin et al. 2004
Point Pattern Analysis (PPA)	No	None	http://www.nku.edu/~longa/cgi-bin/cgi-tcl-examples/generic/ppa/ppa.cgi; http://www-rohan.sdsu.edu/~aldstadt/tools.htm	Alstadt et al. 1998
R-Geo 2.0.0^a^	No	Compatible	http://cran.r-project.org	R Project 2004
S+SpatialStats	Yes	Compatible	http://www.insightful.com	Insightful Corporation 1999
SaTScan^a^	No	None	http://www.satscan.org	Kulldorff and Nagarwalla 1995; SaTScan 2005
SpaceStat	Yes	Compatible	http://www.terraseer.com/products/spacestat.html	Anselin 2001

a Features of these software packages are compared in a review by Anselin (2004).
